# Association Between Postoperative Complications and Real-Time Oxidative Stress Measurements Using Electron Spin Resonance: An Observational Study

**DOI:** 10.7759/cureus.95199

**Published:** 2025-10-22

**Authors:** Shigekiyo Matsumoto, Yoshihide Kuribayashi, Takenori Makino, Shinya Kai, Yoshimasa Oyama

**Affiliations:** 1 Anesthesiology and Intensive Care Medicine, Oita University Faculty of Medicine, Yufu, JPN

**Keywords:** ascorbate, electron spin resonance, oxidative stress, postoperative complications, surgical apgar score

## Abstract

Introduction

Surgical stress induces oxidative stress, which can affect postoperative recovery. Real-time monitoring of oxidative stress can provide valuable insights for perioperative management. This study aimed to investigate the relationship between perioperative oxidative stress and postoperative complications using real-time ascorbyl free radical (AFR) measurements via electron spin resonance (ESR).

Methods

Patients who underwent elective surgery were included in this study. Plasma samples were collected before and after surgery. Using the ESR spectrometer installed in the operating room, the intensity of the plasma AFR levels supplemented with dimethyl sulfoxide (AFR/DMSO) was measured. Clinical parameters, including the surgical Apgar score and National Surgical Quality Improvement Program-defined postoperative complications, were obtained from medical records. This study analyzed the relationship between the AFR/DMSO levels, surgical Apgar score, and postoperative complications. The perioperative factors influencing postoperative AFR/DMSO levels were also investigated.

Results

Postoperative AFR/DMSO levels were significantly lower than the preoperative values (0.26 vs. 0.32, P<0.001). Postoperative AFR/DMSO levels were positively correlated with the surgical Apgar score (r=0.25, P=0.002) and negatively correlated with the number of postoperative complications (r=-0.22, P=0.007). Multivariate analysis identified prolonged surgical duration and elevated postoperative inflammation markers as significant predictors of reduced postoperative AFR/DMSO.

Conclusion

ESR-based AFR/DMSO is a sensitive real-time indicator of oxidative stress and a valuable biomarker for predicting postoperative outcomes.

## Introduction

Surgical stress induces inflammatory responses and oxidative stress. Oxidative stress, arising from an imbalance between oxidants such as reactive oxygen species (ROS) and antioxidants, plays an important role in perioperative biological responses. Indeed, excessive ROS has been reported to cause vascular endothelial injury and organ damage, leading to postoperative complications [1,2]. Therefore, rapid evaluation of oxidative stress during the perioperative period may facilitate postoperative prognosis prediction and therapeutic interventions aimed at controlling oxidative stress [1].

In clinical settings, ROS, such as hydroxyl radicals and superoxide, are extremely unstable, making their direct detection challenging [3]. In contrast, blood antioxidants are relatively stable and can be measured. Ascorbate (vitamin C) is rapidly mobilized against excessive ROS, resulting in faster and more substantial changes in its blood concentration compared to other antioxidants [4]. Consequently, a significant decrease in ascorbate levels indicates higher oxidative stress, making ascorbate a sensitive biomarker for oxidative stress. However, high-performance liquid chromatography (HPLC), the most reliable method for measuring ascorbate, is time-consuming, rendering it unsuitable for real-time evaluation. To overcome this limitation, this study focused on plasma ascorbyl free radicals (AFR), which can be measured within 12 min of blood sampling using an electron spin resonance (ESR) device installed in the operating room. Our previous studies demonstrated a correlation between plasma ascorbate concentrations measured by HPLC and AFR signals detected upon the addition of dimethyl sulfoxide (DMSO) to plasma (AFR/DMSO). These findings suggest that the AFR/DMSO measurement could serve as a real-time indicator of plasma ascorbate levels and oxidative stress [5].

Plasma AFR/DMSO levels were measured in real time using an ESR device before and after elective surgery, and the relationship between AFR/DMSO levels and postoperative prognostic indicators was evaluated. This study used the widely recognized surgical Apgar score (SAS) and postoperative complications as defined by the National Surgical Quality Improvement Program (NSQIP) as postoperative prognostic indicators [6-8]. The primary endpoint was the relationship between plasma AFR/DMSO levels, the SAS, and the NSQIP-defined postoperative complications. As a secondary endpoint, the perioperative factors influencing postoperative AFR/DMSO levels were investigated.

## Materials and methods

Patients and data collection

This prospective observational study was approved by the Ethics Committee of the Oita University Faculty of Medicine (approval number: 748). The study included patients aged ≥20 years who underwent elective surgery at Oita University Hospital between August 25 and September 26, 2014, and required the insertion of an arterial catheter for continuous arterial pressure monitoring during surgery. Pregnant women and those who had undergone cardiopulmonary bypass surgery were excluded from the study. Written informed consent was obtained from all participants before enrollment.

Patient demographic data, including age, sex, body mass index (BMI), preoperative comorbidities (heart disease, myocardial infarction (MI), congestive heart failure (CHF), angina, coronary revascularization, pulmonary disease, pneumonia, chronic obstructive pulmonary disease (COPD), and dyspnea), diabetes mellitus, hypertension, and intraoperative factors (type of surgery performed, surgery time, anesthesia time, fluid infusion volume, urinary volume, transfusion volume, and estimated blood loss) were collected from electronic medical records. Laboratory data (aspartate aminotransferase (AST), alanine aminotransferase (ALT), blood urea nitrogen (BUN), creatinine, and C-reactive protein (CRP) levels) were collected preoperatively and on postoperative day 1. Intraoperative vital signs were analyzed every minute to determine the lowest mean arterial pressure and heart rate. The SAS was calculated based on the lowest mean arterial pressure, the lowest heart rate, and the estimated blood loss during surgery according to the criteria in Table 1 [8].

**Table 1:** Surgical Apgar Score.

Parameters	Number of points				
	0	1	2	3	4
Blood loss (mL)	>1,000	601-1,000	101-600	≦100	
Lowest mean arterial blood pressure (mmHg)	<40	40-54	55-69	≧70	
Lowest heart rate (beats/min)	>85	76-85	66-75	56-65	≦55

Outcomes

The primary objective was to evaluate the relationship among plasma AFR/DMSO levels, the SAS, and the NSQIP-defined postoperative complications. A secondary objective was to investigate the perioperative factors that influence postoperative AFR/DMSO levels.

AFR/DMSO measurement

An arterial catheter was inserted preoperatively for continuous blood pressure monitoring. A 1.0 mL arterial blood sample was collected through the catheter into a heparinized syringe designed for blood gas analysis (SafePICO Aspirator syringe; Radiometer, Copenhagen, Denmark). Immediately after collection, blood samples were centrifuged (3000 g, 4°C, for 10 minutes) to collect plasma. Blood samples were collected before and immediately after surgery in the operating room.

**Figure 1:**
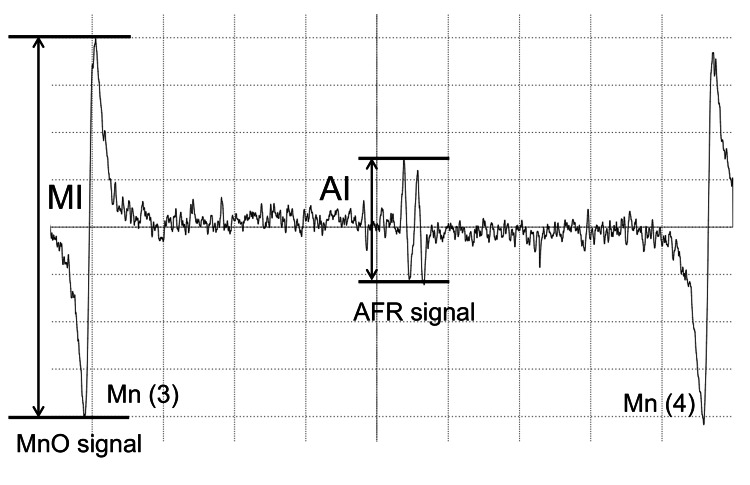
AFR and MnO ESR spectra. Typical ESR signal of the AFR in DMSO-supplemented plasma. The signal of the plasma AFR doublet is specified. The relative height of the first AFR line against an internal standard, Mn (3), is defined as AFR/DMSO. Plasma AFR/DMSO=AI/MI. Abbreviations: AFR: ascorbyl free radical; DMSO: dimethyl sulfoxide; MI: manganese oxide signal intensity; AI: ascorbyl free radical signal intensity.

The AFR/DMSO measurements were made according to previously described methods [5]. Within two minutes after centrifugation, 50 µL of plasma was transferred to a new light-shielding polyethylene tube and mixed with 100 µL of DMSO. The mixture was stirred for 10 seconds and then aspirated into a quartz cell for the ESR measurement. ESR signals were measured using a JES-FR30 spectrometer (JEOL Ltd., Tokyo, Japan), employing manganese oxide as an internal standard marker of the ESR cavity, which exhibits six distinct signals at room temperature. The AFR/DMSO signals were measured between the third and fourth manganese oxide signals, appearing as two peaks of nearly identical intensity. The intensity of the AFR signal relative to the third manganese oxide signal was calculated. This was determined to be the plasma AFR/DMSO value (Figure 1).

Postoperative outcomes

Postoperative complications defined using the NSQIP were considered major complications [7]. NSQIP-defined complications within 30 days after surgery include death, acute renal failure, bleeding requiring four or more units of red blood cell transfusion within 72 hours post-surgery, cardiac arrest requiring cardiopulmonary resuscitation, coma lasting ≥24 hours, deep venous thrombosis, MI, unplanned intubation, ventilator use ≥48 hours, pneumonia, pulmonary embolism, stroke, wound disruption, deep or organ-space surgical site infection, sepsis, septic shock, systemic inflammatory response syndrome (SIRS), and vascular graft failure. Complications were identified from the electronic medical records, and the total number of complications per patient was calculated.

Sample size calculation

The sample size was determined using IBM SPSS Statistics version 28.0 (IBM, Armonk, NY, USA), targeting the primary endpoint: the correlation between postoperative AFR/DMSO and postoperative complications. Assuming a two-sided α of 0.05, a power (1-β) of 0.8, and an effect size of 0.25, the required sample size was calculated to be 128 participants.

Statistical analysis

Statistical analyses were performed using IBM SPSS Statistics version 28.0 (IBM), with statistical significance set at P<0.05. Data normality was assessed using the Shapiro-Wilk test. Continuous variables were expressed as medians and interquartile ranges (IQR), while categorical variables were presented as counts and percentages. The AFR/DMSO levels before and after surgery were compared using paired t-tests, and other laboratory data were compared using the Wilcoxon signed-rank test. Correlations between postoperative AFR/DMSO levels and patient, intraoperative, and postoperative factors were analyzed using Spearman's rank correlation. Additionally, multivariate linear regression analysis was performed to identify the factors influencing postoperative AFR/DMSO levels. Age was forced into the model, and other significant factors identified in the univariate analysis (Spearman's correlation) were selected using a forward stepwise method.

## Results

Patient characteristics

**Figure 2:**
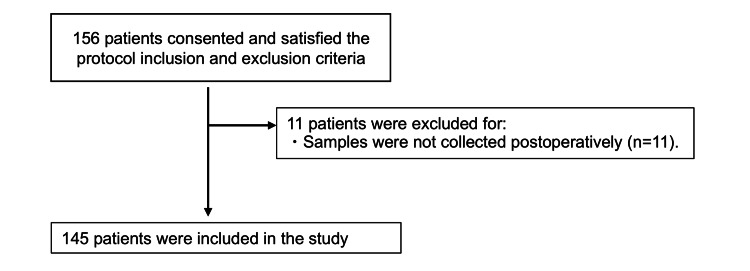
Flow diagram showing patient enrollment and analysis.

Written informed consent was obtained from all 156 patients. Of these, 11 patients with missing laboratory data on postoperative day 1 were excluded, leaving 145 patients for analysis (Figure 2). The patient demographics and surgical data are shown in Table 2. The median patient age was 67 years (IQR: 58-76 years), and 62 patients (43%) were women. Abdominal surgery was the most common (56 cases, 39%), followed by orthopedic surgery (33 cases, 23%) and thoracic surgery (19 cases, 13%). Median surgery time was 3.8 hours (IQR: 2.5-5.9 hours), median estimated blood loss was 120 mL (IQR: 20-400 mL), and transfusions were administered to 31 patients (21%).

**Table 2:** Patient Characteristics (n=145). Abbreviations: MI, Myocardial infarction; CHF, chronic heart failure; COPD, chronic pulmonary disease The data are presented as median (interquartile range) or n (%).

Patient characteristics (n=145)		Values
Preoperative characteristics		
	Age (years)	67 (58-76)
	Sex (Female/Male)	62/83
	Body mass index	23.2 (20.7-25.1)
	Underlying disease	
	Cardiovascular disease (MI, CHF, angina, coronary revascularization)	18 (12%)
	Pulmonary disease (pneumonia, COPD, dyspnea)	17 (12%)
	diabetes mellitus	23 (16%)
	Hypertension	45 (31%)
Types of surgery		
	Cardiovascular	12 (8%)
	Thoracic	19 (13%)
	Abdominal	56 (39%)
	Neurosurgery	8 (6%)
	Orthopedic	33 (23%)
	Brest/skin/soft tissue	17 (12%)
Intraoperative characteristics		
	Surgery time (hour)	3.8 (2.6-5.9)
	Infusion (mL)	2050 (1350-2900)
	Estimated blood loss (mL)	120 (20-400)
	Transfusion, n(%)	31 (21%)
	Transfusion (mL)	0 (0-0)
	Urine (mL)	260 (180-560)

Changes in AFR/DMSO levels and laboratory data

**Figure 3:**
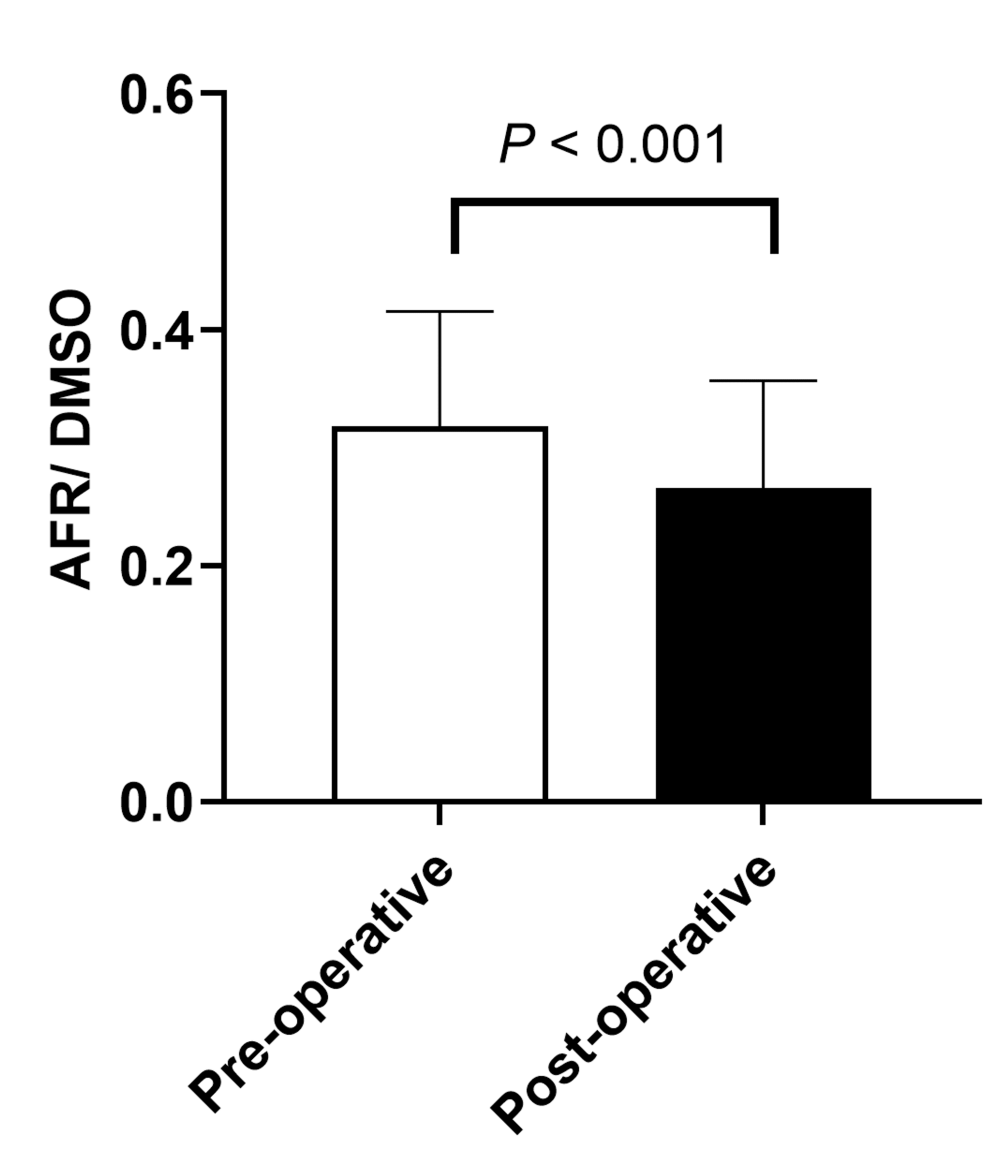
Changes in AFR/DMSO. The plasma samples were obtained before and after the surgery. Plasma AFR/DMSO was measured using an ESR installed in the operating room. Abbreviations: AFR, Ascorbyl free radical; ESR, electron spin resonance; DMSO, dimethyl sulfoxide

AFR/DMSO significantly decreased postoperatively (median 0.26, IQR 0.20-0.34) compared to preoperative values (median 0.32, IQR 0.26-0.38; P<0.001, Figure 3). CRP levels significantly increased on postoperative day 1, along with significant changes in AST, BUN, and creatinine levels (Table 3).

**Table 3:** Alterations in Evaluated Laboratory Parameters. Abbreviations: AST, aspartate aminotransferase; ALT, alanine aminotransferase; BUN, blood urea nitrogen; CRP, C-reactive protein. Data are median (interquartile range).

	Preoperative	Postoperative (24h after surgery)	P value
AST (U/L)	20 (17-27)	24 (19-36)	<0.001
ALT (U/L)	16 (11-25)	15 (11-26)	0.84
BUN (mmol/L)	5.4 (4.4-6.4)	4.5 (3.5-6.2)	<0.001
Creatinine (µmol/L)	69.9 (56.6-84.0)	68.1 (53.0-83.1)	<0.001
CRP (mg/L)	1.1 (0.4-3.0)	37.5 (21.1-61.7)	<0.001

Relationship between postoperative AFR/DMSO levels and postoperative outcomes

**Figure 4:**
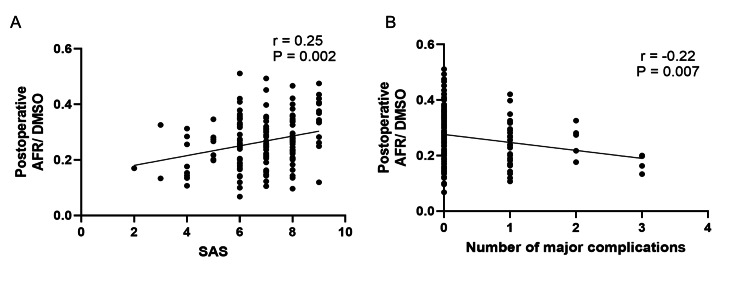
Correlation between postoperative AFR/DMSO and SAS or postoperative complications. (A) Correlation between postoperative AFR/DMSO and SAS. (B)Correlation between postoperative AFR/DMSO and NSQIP-defined postoperative complications. Abbreviation: SAS, surgical apgar score

The median SAS was 7 (IQR, 6-8). At least one NSQIP-defined major complication occurred in 39 patients (27%), 31 of whom were classified as having SIRS (Table 4). Postoperative AFR/DMSO values showed a significant positive correlation with SAS (r=0.25, P=0.002, Figure 4A) and a significant negative correlation with the number of NSQIP-defined postoperative major complications(r=-0.22, P=0.007, Figure 4B).

**Table 4:** SAS and Postoperative Complications. Abbreviation: SAS, surgical Apgar score The data are median (interquartile range) or n (%). National Surgical Quality Improvement Program (NSQIP)-defined events were considered major complications.

Postoperative outcomes	Values
SAS	7 (6-8)
Number of major complications >1, n (%)	39 (27%)
Number of major complications	0 (0-0)

Factors influencing postoperative AFR/DMSO levels

Postoperative AFR/DMSO positively correlated with preoperative AFR/DMSO (P<0.001) and negatively correlated with surgery time (P<0.001), fluid volume (P<0.001), urinary volume (P=0.001), estimated blood loss (P<0.001), postoperative BUN (P=0.016), and postoperative CRP levels (P<0.001). Multivariate regression analysis revealed that the preoperative AFR/DMSO (P<0.001), surgery time (P<0.001), and postoperative CRP level (P<0.001) were significant independent predictors of the postoperative AFR/DMSO (Table 5).

**Table 5:** Univariate and Multivariate Linear Regression Analysis. Abbreviations: CI, Confidence interval; AST, aspartate aminotransferase; ALT, alanine aminotransferase; BUN, blood urea nitrogen; CRP, C-reactive protein

Variables	Univariate analysis		Multivariate analysis	
	Coefficient (r)	P-value	β coefficient (95%CI)	P-value
Age	-0.131	0.118	0.00(-0.001 to 0.000)	0.211
Preoperative AFR/DMSO	0.689	<0.001	0.62 (0.53-0.70)	<0.001
Surgery time (hour)	-0.48	<0.001	-0.013 (-0.017 to -0.010)	<0.001
Infusion (ml)	-0.354	<0.001	-	
Urine (ml)	-0.275	0.001	-	
Estimated blood loss (ml)	-0.366	<0.001	-	
Postoperative AST (U/L)	-0.044	0.602	-	
Postoperative ALT (U/L)	0.091	0.275	-	
Postoperative BUN (mmol/L)	-0.2	0.016	-	
Postoperative Creatine (µmol/L)	-0.135	0.104	-	
Postoperative CRP (mg/L)	-0.369	<0.001	-0.001 (-0.001 to 0.000)	<0.001

## Discussion

It is known that perioperative oxidative stress may increase the risk of postoperative complications and delay postoperative recovery [1,9,10]. This present study focused on ascorbate, which is highly sensitive to oxidative stress, to evaluate perioperative oxidative stress. Previously, it was reported that ascorbate can be measured as AFR/DMSO using an ESR device, enabling rapid assessment shortly after sample collection [5]. This study measured AFR/DMSO values pre- and postoperatively in the operating room, revealing significant correlations between postoperative AFR/DMSO levels and both postoperative complications and SAS scores.

An ESR device was utilized to measure the AFR/DMSO values for the real-time evaluation of oxidative stress. Conventionally, oxidative stress has been assessed using markers such as malondialdehyde, 4-hydroxy-nonenal, and F2-isoprostane 15(S)-8-iso-prostaglandin F2α, which reflect the lipid peroxidation of cell membranes caused by free radicals [11,12]. These conventional oxidative stress markers represent accumulated damage, which complicates the detection of rapid changes and poses technical challenges for real-time measurements [13]. In contrast, AFR/DMSO measurement using ESR provides a rapid assessment of ascorbate oxidation status, allowing swift and sensitive tracking of perioperative oxidative stress fluctuations [5]. Our study demonstrated a significant decrease in postoperative AFR/DMSO values compared to preoperative values, measured in real time by ESR immediately upon sample collection. Reduced postoperative AFR/DMSO values were associated with prolonged surgery and elevated postoperative CRP levels, suggesting that extended and highly invasive surgeries triggering inflammatory responses may elevate oxidative stress and rapidly deplete ascorbate levels.

This study evaluated postoperative risk factors using the SAS and examined the number of postoperative complications defined by the NSQIP, as well as their correlations with postoperative AFR/DMSO values. Complications defined in the NSQIP database are widely used for postoperative prognostic evaluation, and the occurrence of postoperative complications is known to affect long-term outcomes negatively. Indeed, it has been reported that postoperative complications affect long-term prognosis more than preoperative patient risk factors [6,7]. The SAS, calculated from three intraoperative parameters - the lowest mean arterial pressure, the lowest heart rate, and estimated blood loss - is useful for assessing the risk of postoperative complications and has been correlated with postoperative complications defined by the NSQIP [14-16]. This present study’s results revealed a significant positive correlation between postoperative AFR/DMSO values and SAS, as well as a negative correlation with the number of NSQIP-defined postoperative complications.

Previous studies have also reported associations between perioperative oxidative stress assessed using derivatives of reactive oxygen metabolites (d-ROMs) and postoperative inflammation, delayed postoperative recovery, postoperative complications [10,17], and postoperative delirium [18]. Consistent with previous reports, this study demonstrated that elevated postoperative CRP levels correlated with rapidly decreasing AFR/DMSO values after surgery, suggesting that AFR/DMSO has the potential to predict postoperative inflammatory responses. These findings suggest that postoperative AFR/DMSO is not only a sensitive marker of oxidative stress but may also be a valuable biomarker for predicting postoperative outcomes.

In this study, lower AFR/DMSO levels were correlated with postoperative complications. Although animal studies indicate that ascorbate attenuates ischemia-reperfusion injury and protects against organ failure [19, 20], clinical studies have not conclusively shown that ascorbate supplementation improves postoperative outcomes [21-24]. These discrepancies may be attributed to the lack of personalized supplementation based on individual ascorbate levels. Oxidative stress is defined as an imbalance between oxidants and antioxidants [25]. Excessive antioxidant administration may paradoxically induce DNA damage via free radicals [26]. Indeed, animal studies have reported the exacerbation of myocardial oxidative stress upon the administration of vitamin E or ascorbate to healthy pigs [27], and meta-analyses including healthy individuals have indicated an increased mortality associated with antioxidant supplementation [28]. These findings highlight the importance of maintaining an oxidant-antioxidant balance in oxidative stress management, indicating that antioxidant supplementation should be targeted toward patients with antioxidant deficiencies. The ESR-based AFR/DMSO measurement employed in this study enables rapid, real-time assessment of perioperative oxidative stress, potentially allowing timely ascorbate supplementation interventions through a point-of-care system.

This study had several limitations. First, the single-center, short-term observational design and limited sample size restrict the generalizability of the present findings. More extensive multicenter studies are necessary to confirm these findings. Second, the correlation between the AFR/DMSO levels and long-term postoperative outcomes was not examined, necessitating further research to evaluate the potential of the AFR/DMSO as a long-term prognostic indicator. Finally, the feasibility of measuring AFR/DMSO is limited by the availability of ESR equipment.

## Conclusions

This study validates that ESR-based AFR/DMSO measurement provides a sensitive real-time assessment of perioperative oxidative stress. We found that reduced postoperative AFR/DMSO values, indicative of rapid antioxidant consumption, were associated with prolonged surgery and elevated postoperative CRP levels, suggesting a direct link between surgical invasiveness and increased oxidative burden. Crucially, the immediate postoperative AFR/DMSO level demonstrated significant correlation with both the SAS and the number of postoperative complications. These findings establish AFR/DMSO as a valuable prognostic biomarker with the potential to guide the optimization of individualized ascorbate supplementation therapy in perioperative patient management.
